# A Role for Neutral Sphingomyelinase in Wound Healing Induced by Keratinocyte Proliferation upon 1*α*, 25-Dihydroxyvitamin D_3_ Treatment

**DOI:** 10.3390/ijms20153634

**Published:** 2019-07-25

**Authors:** Federica Filomena Patria, Maria Rachele Ceccarini, Michela Codini, Carmela Conte, Luana Perioli, Tommaso Beccari, Elisabetta Albi

**Affiliations:** Department of Pharmaceutical Science, University of Perugia, 06126 Perugia, Italy

**Keywords:** cell proliferation, keratinocyte, neutral sphingomyelinase, vitamin D, wound healing repair

## Abstract

The skin has many functions, such as providing a barrier against injury and pathogens, protecting from ultraviolet light, and regulating body temperature. Mechanical causes and many different pathologies can lead to skin damage. Therefore, it is important for the skin to be always adaptable and renewable and for cells to undergo proliferation. Here, we demonstrate that 1*α*, 25-dihydroxyvitamin D_3_ (VD3) stimulates keratinocyte proliferation, leading to wound closure in a simulation model of injury. Functionally, our results show that VD3 acts by stimulating cyclin D1, a cyclin that promotes the G1/S transition of the cell cycle. The study on the mechanism underlying cyclin D1 expression upon VD3 stimulation clearly demonstrates a key role of neutral sphingomyelinase. The enzyme, whose gene and protein expression is stimulated by VD3, is itself able to induce effects on cyclin D1 and wound healing similar to those obtained with VD3. These results could be very useful in the future to better understand wound mechanisms and improve therapeutic interventions.

## 1. Introduction

The epidermis, derived from the ectoderm, is a keratinized stratified squamous epithelium that rests on the dermis, which houses skin appendages (sweat glands and hairs), many sensory neurons, and blood vessels. The dermis, in turn, is placed above the hypodermis, the deepest layer of the skin [1]. Thus, the epidermis is the largest epithelium of the whole body as it covers its entire external surface. The epidermis participates in the important functions of the skin, such as providing a mechanical barrier against injury and pathogens, protecting from UV light, and regulating body temperature. The epithelium includes different layers which, from inside to outside, are basale, spinosum, granulosum, lucidum, and corneum stratum. All strata are composed of different cell types: melanocytes, which derive from neural crest cells and produce melanin; Langerhans cells, which are of mesenchymal origin and are antigen-presenting cells; Merkel cells, which are modified epidermal cells that have sensory function for fine-touch; and keratinocytes, which represent the predominant cell type of epidermis. Keratinocytes originate in the basal stratum, where they are fed by dermis. In the basal stratum, keratinocytes have low keratinization. They then move toward different strata up to the most superficial stratum, where the cells, which are practically composed of only keratin and are completely free of water, are lost. Keratinocytes have always been considered as cells that are able to produce keratin and to provide only physical support to intraepidermal nerve fibers. However, in actual fact, they contribute to somatosensation by releasing glutamate [2] and acetylcholine [3] or ATP [4] and can thus transduce nociceptive responses [5]. 

The epidermis, and therefore keratinocites, have the potential risk of being exposed to traumatic damage; genotoxic stress, including chemical compounds; ionizing radiation; ultraviolet rays [6]; and inflammatory agents [7], or having severe cutaneous adverse reactions to drugs [8].

The skin represents both the site of 1*α*,25-dihydroxyvitamin D_3_ (VD3) synthesis and a target tissue for its biologically active form. VD3 is a secosteroid, which was initially known for its skeletal role but which is now also known for its numerous pleiotropic effects. In fact, VD3 is important for protection against skin inflammation [9] and is used as an important therapeutic option in the treatment of psoriasis [10]. VD3 plays an important role in the keratinocyte differentiation process by regulating the sequential turning on and off of the genes producing the elements required for differentiation as well as activating those enzymes involved in differentiation [11].

In addition, the protective effects of VD3 derivatives against UVB-induced damage in human keratinocytes have been described [12]. Interestingly, a reduction in proliferation of keratinocytes in the dermal portion of the hair follicle in vitamin D receptor (VDR) null mice was demonstrated [13].

Growing evidence established over the last two decades shows that VD3 induces a regulated modulation of bioactive sphingolipids [14]. Among these molecules, sphingomyelin (SM) [15], neutral sphingomyelinase (nSMase) [16], ceramide [17], and sphingosine-1-phosphate (S1P) [18] are involved in the mechanism of VD3 action.

To address the need for a comprehensive analysis of the role of VD3 in keratinocyte proliferation, we set out to simulate wound heading in vitro. In addition, the VD3-nSMase cross-talk was studied by analyzing specific gene and protein expression. Here, we show for the first time that nSMase is a key target for the proliferative stimulus induced by VD3 in keratinocytes after simulating the induction of a wound. The results demonstrate a specific link between VD3 and nSMase and the role of VD3 in keratinocyte proliferation in wound healing.

## 2. Results

### 2.1. At Normal Cell Density, Keratinocytes Remain Vital and Increase in Cell Volume with Physiological and High Doses of VD3

**Figure 1:**
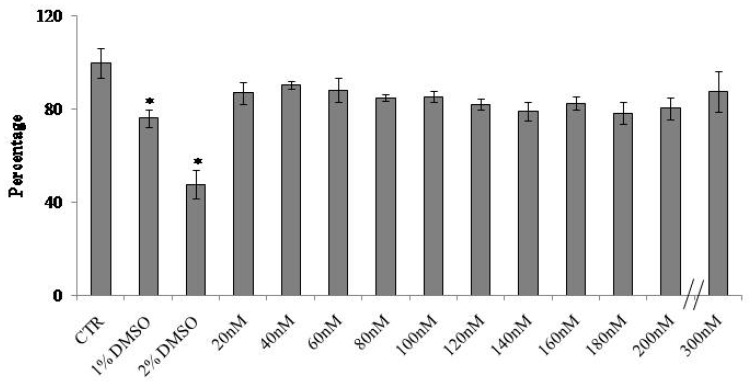
Effect of 25-dihydroxyvitamin D_3_ (VD3) on HaCaT cell viability. Cells were cultured with increasing doses of VD3 from 20 to 300 nM for 24 h, and the viability was measured by MTT assay. Values are reported as percentage viability of the control sample; 1% DMSO and 2% DMSO were used as positive controls. Data are expressed as mean ± SD of three independent experiments performed in duplicate. Significance, * *p* < 0.001 versus the control sample.

**Figure 2:**
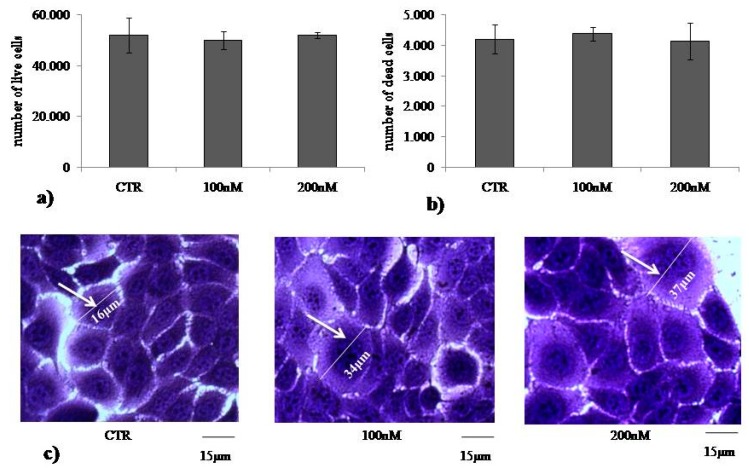
Effect of VD3 on HaCaT cell number and morphology. Cells were cultured in the presence of 100 and 200 nM VD3 for 24 h and then counted ((**a**) live cells) or submitted to trypan blue exclusion assay ((**b**) dead cells) or treated with stained solution of wound healing assay ((**c**) cell morphology). For (**a**) and (**b**), data are expressed as mean ± SD of three independent experiments performed in duplicate. Significance, * *p* < 0.001 versus control sample.

To study the role of VD3 in regulating the homeostasis of the epidermis, we used immortalized human HaCaT keratinocytes. First, we set out to identify the dose-dependent effect of VD3 on cell viability after 24 h of culture using MTT assay. The results showed that dosage from 20 to 300 nM VD3 induced more than 80% cell viability with no significant changes at all concentrations used; 1% and 2% DMSO were used as positive controls (CTR) (Figure 1). Considering that HaCaT is an immortalized cell line, the slight reduction of cell viability at all VD3 concentrations compared to the control is normal, and the standard deviation conforms to the experimental system [19]. Thus, VD3 did not induce cytotoxicity. As 100 nM is the physiological concentration of VD3 [20] and our results showed that 200 nM VD3 did not change cell viability with respect to 100 nM VD3, we decided to use 100 and 200 nM VD3 for all the following experiments. The HaCaT cell number treated with both concentrations was similar to the control (Figure 2a). Trypan blue exclusion assay confirmed good cell viability, with 8% of dead cells in both CTR and experimental cells (100 and 200 nM VD3, Figure 2b). The morphological analysis showed that HaCaT cells had a fairly uniform volume (average diameter of 15.5 ± 1.3 μm) in the control sample. Treatment with VD3 induced an increase in the volume of some cells, equal to about twice that of the control cells (Figure 2c).

### 2.2. VD3 Promotes Keratinocite Proliferation in Wound Healing Simulation

**Figure 3:**
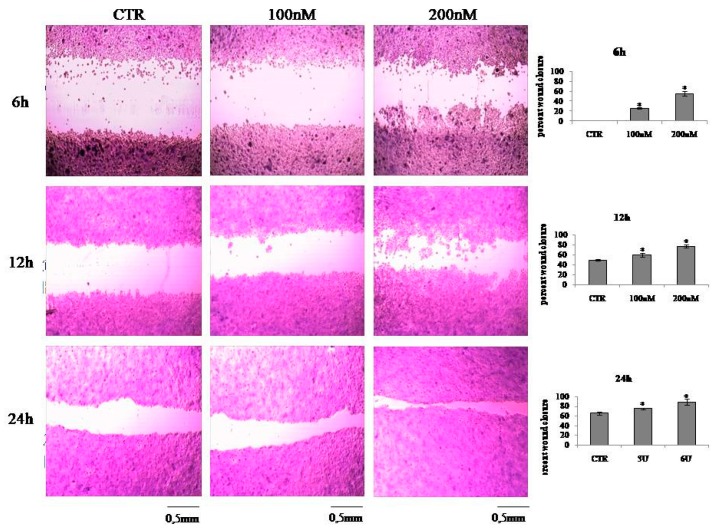
Effect of VD3 in wound closure. HaCaT cells were treated with 100 and 200 nM VD3, and wound healing assay was performed after 6, 12, and 24 h. The left figure is the panel of microscopy images, and the right figure shows the percentage of closure. Data are expressed as mean ± SD of three independent experiments performed in duplicate. Significance, * *p* < 0.001 versus control sample.

To test the hypothesis that VD3 can stimulate keratinocyte proliferation following induced damage, we used the wound healing test. An open gap of 0.9 mm was created by a “wound” according to the manufacture’s instruction, and the reaction of the cells to fill the damaged area was inspected microscopically over time (6, 12, and 24 h). In the CTR sample, the open gap remained unchanged after 6 h and reduced to 0.45 ± 0.03 mm and 0.3 ± 0.02 mm after 12 h and 24 h, respectively (Figure 3). It was evident that VD3 induced a strong “healing” effect. In fact, the open gap reduced to 0.67 ± 0.05 mm with 100 nM VD3 and to 0.45 ± 0.03 mm with 200 nM VD3 after only 6 h. After 12 h, the open gap reduced to 0.36 ± 0.04 mm with 100 nM VD3 and to 0.22 ± 0.03 mm with 200 nM VD3. After 24 h, the open gap reduced to 0.23 ± 0.01 mm with 100 nM VD3 and to 0.11 ± 0.02 mm with 200 nM VD3 (Figure 3).

**Figure 4:**
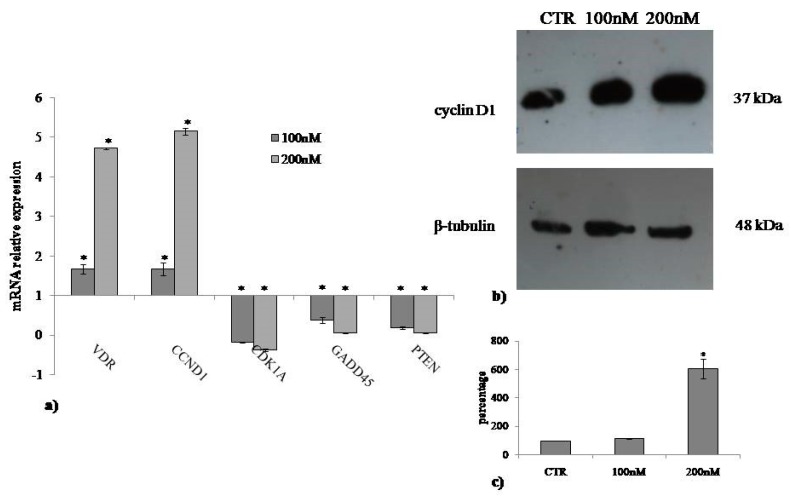
Effect of VD3 on cyclin D1. (**a**) Gene expression evaluated by RT-PCR; (**b**) immunoblotting, with the position of the 37 kDa protein for cyclin D1 and 48 kDa for β-tubulin indicated in relation to the position of molecular size standards; (**c**) area density evaluated by densitometry scanning and analyzed with ImageJ program. Values of cyclin D1, normalized with β-tubulin, are expressed as fold changes with respect to the control sample, which is considered equal to 1. Significance, * *p* < 0.001 versus control sample.

Many reports have shown that the response of VD3 is identified by the gene expression of its receptor (VDR) [21,22]. Thus, to highlight the molecular response of HaCaT to VD3 treatment, we analyzed the VDR gene expression. The results showed upregulation of VDR, confirming the molecular response of the cells after 24 h of culture. Next, we tested the hypothesis that VD3 can induce wound closure by regulating the cell cycle. For this, we performed the analysis of cyclin D1 encoded by the *CCND1* gene and cyclin-dependent kinase inhibitor 1A (CDK1A) encoded by the homonymous gene. To expand the study on proliferation, *GGDD45*, a molecule involved in cell growth arrest, and the oncosuppressor *PTEN* were analyzed. As shown in Figure 4, upregulation of *CCDN1* and downregulation of *CDK1*, *GGDD45*, and *PTEN* were observed. The increase in cyclin D1 protein was demonstrated by immunoblotting analysis. 

### 2.3. Involvement of Neutral Sphingomyelinase in VD3-induced Cell Proliferation 

**Figure 5:**
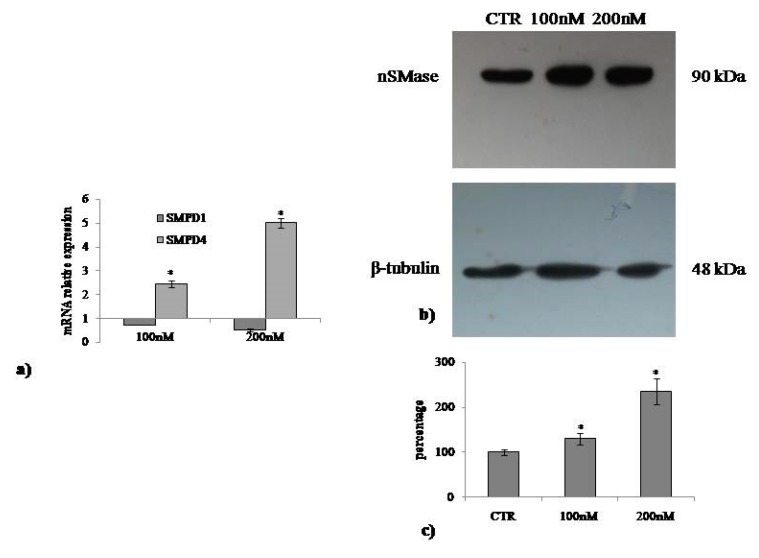
Effect of VD3 on nSMase. (**a**) Gene expression evaluated by RT-PCR; (**b**) immunoblotting, with the position of the 90 kDa protein for nSMase and 48 kDa for β-tubulin indicated in relation to the position of molecular size standards; (**c**) area density evaluated by densitometry scanning and analyzed with ImageJ program. Values of nSMase, normalized with β-tubulin, are expressed as fold changes with respect to the control sample, which is considered equal to 1. Significance, * *p* < 0.001 versus control sample.

**Figure 6:**
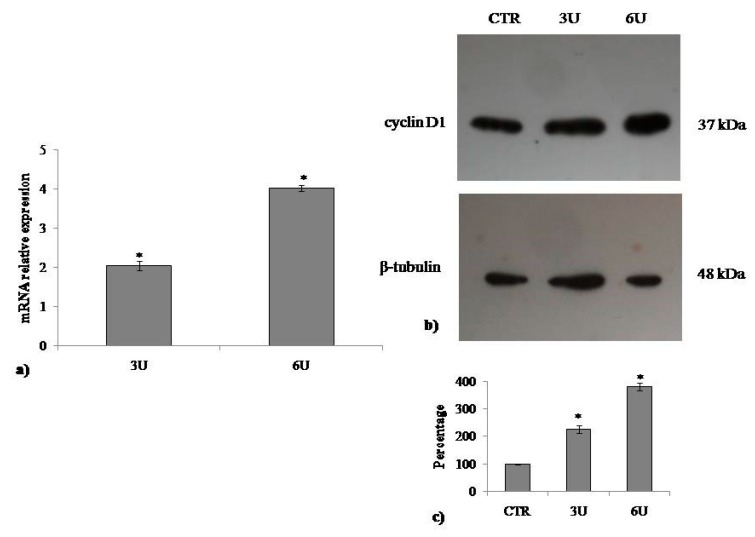
Effect of nSMase on cyclin D1. (**a**) Gene expression evaluated by RT-PCR; (**b**) immunoblotting, with the position of the 37 kDa protein for cyclin D1 and 48 kDa for β-tubulin indicated in relation to the position of molecular size standards; (**c**) area density evaluated by densitometry scanning and analyzed with ImageJ program. Values of cyclin D1, normalized with β-tubulin, are expressed as fold changes with respect to the control sample, which is considered equal to 1. Significance, * *p* < 0.001 versus control sample.

**Figure 7:**
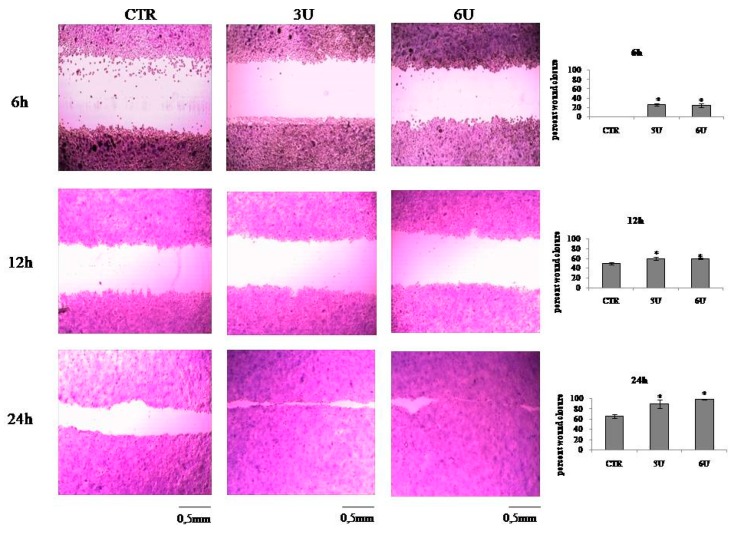
Effect of nSMase in wound closure. HaCaT cells were treated with 3U and 6U nSMase, and wound healing assay was performed after 6, 12, and 24 h. The left figure shows the panel of microscopy images. The right figure shows the percentage of closure. Data are expressed as mean ± SD of three independent experiments performed in duplicate. Significance, * *p* < 0.001 versus control sample.

The above results indicate that VD3 stimulates cell proliferation. nSMase has been implicated as a mediator of the actions of VD3 in cells [23,24]. To assess its role in the effect of VD3 in HaCaT cell proliferation, *SMPD4* and *SMPD1* gene expression was evaluated after 24 h from VD3 treatment. The specific effectiveness of the VD3 on *SMPD4* gene upregulation was demonstrated (Figure 5a). Next, the increase in nSMase protein was demonstrated by immunoblotting analysis (Figure 5b). To determine whether the findings in the experimental model are relevant for the role of VD3 in the cell cycle, we cultured HaCaT cells with 3U or 6U of nSMase and studied the gene and protein expression of cyclin D1. Figure 6 demonstrates that CCDN1 was upregulated and cyclin D1 was overexpressed compared to the CTR sample in an nSMase dose-dependent manner. Therefore, we sought to examine whether nSMase was able to stimulate the wound healing process. Notably, nSMase stimulated wound closure (Figure 7). In the CTR sample, the values were the same as the above reported experiments (Figure 7). It was evident that nSMase induced a strong “healing” effect. In fact, the open gap reduced to 0.67 ± 0.02 mm with 3U nSMase and to 0.58 ± 0.04 mm with 6U nSMase after only 6 h. After 12 h, the open gap diminished to 0.40 ± 0.01 mm with 3U nSMase and to 0.40 ± 0.03 mm with 6U nSMase. After 24 h, the open gap reduced to 0.09 ± 0.01 mm with 3U nSMase and to 0.009 ± 0.0008 mm with 6U nSMase (Figure 7).

## 3. Discussion

VD3 has been suggested to function as a putative multitargeting molecule because of its ability to have extraskeletal effects in many tissues, including the skin [25]. It has been reported that the expression of carbonic anhydrase II and IX are significantly upregulated in keratinocytes by VD3 treatment [26]. In addition, VD3 is useful for psoriasis treatment [27]. Previously, Hill et al. [28] demonstrated that VD3 regulates keratinocytes proliferation. The authors highlighted that low doses of VD3 (10 nM) led to an increase in keratinocyte proliferation and that high doses of VD3 (100 nM) resulted in reduced proliferation after 24 h of treatment. Here, we showed that after 24 h, concentration from 20 to 300 nM VD3 did not induce changes in cellular viability, in the number of live and dead cells. Therefore, we used 100 and 200 nM concentrations. A VD3 concentration of 100 nM corresponds to about 38.4 ng/mL, while 200 nM corresponds to about 76.8 ng/mL. Considering that the physiological range of VD3 in the blood is 30–100 ng/mL, both doses that we used represent a physiological concentration of VD3, as previously reported [21]. All the results obtained were positive with 100 nM VD3 concentration but were more strongly evident with 200 nM VD3 concentration, indicating that the average physiological values of VD3 are better than the values close to the physiological minimum limit. We demonstrated that, after 24 h of treatment, VD3 increased keratinocyte cell volume by upregulating the *CCND1* gene and increasing the expression of its encoded cyclin D1 protein. It is known that cyclin D1 is a key regulatory subunit of the holoenzyme that promotes the G1/S phase transition of the cell cycle by phosphorylating the pRB protein [29]. The major goal of this study was to investigate the mechanism underlying cyclin D1 expression upon VD3 stimulation. The contribution of nSMase in the regulation of VD3 function has been well described [16]. Given that nSMase enzyme increases during G1/S transition of the cell cycle by regulating DNA synthesis [30] and gene expression [31], it was important to establish the role of nSMase in cyclin D1 expression in keratinocites. The results showed that VD3 specifically stimulated nSMase gene and protein expression without the involvement of aSMase. Incubation of HaCaT cells with nSMase resulted in cyclin D1 gene and protein overexpression, same as what occurred with VD3. Therefore, our results strongly suggest that VD3 stimulates keratinocyte proliferation via nSMase. Interestingly, in a wound simulation system, both VD3 and nSMase intensively facilitated wound closure with respect to the control.

## 4. Materials and Methods

### 4.1. Materials

Dulbecco’s modified Eagle medium (DMEM), L-glutamine, trypsin, and ethylenediaminetetraacetic acid disodium and tetra-sodium salt (EDTA) were from MicrotechSrl (Pozzuoli, NA, Italy). Fetal bovine serum (FBS), penicillin–streptomycin, and 6X loading dye were obtained from Thermo Fisher Scientific (Waltham, MA, USA). Antibiotics, Dulbecco’s phosphate buffer saline (PBS) pH 7.4, and agarose were from Invitrogen Srl (Milan, Italy). Dimethylsulfoxide (DMSO), ethanol, hydrochloric acid, sodium chloride, and sodium hydroxide were purchased from Carlo Erba ReagentiSrl (Milan, Italy). Trypan blue solution 0.4%, ethidiumbromide, formaldehyde, solution tris(hydroxymethyl)aminimethane (Tris), 3-[4,5-dimethyl-2-thiazolyl]-2,5-diphenyl-2-tetrazoliumbromide (MTT), VD3, and nSMase were obtained from Sigma-Aldrich (now Merck, Darmstadt, Germany). Fixing solution and cell stain solution were from Cell Biolabs, INC (San Diego, CA, USA). RNAqueous^®^-4PCR kit was from Ambion Inc. (Austin, TX, USA). Reverse Transcription kit, TaqMan™ Gene Expression Master Mix, and TaqMan™ Gene Expression Assay were purchased from Applied Biosystems (Foster City, CA, USA). 

### 4.2. Cell Culture and Treatments

Human keratinocytes (HaCaT cell lines) were purchased from I.Z.S.L.E.R. from the Istituto Zooprofilattico Sperimentale della Lombardia e dell’Emilia Romagna ‘Bruno Ubertini’ (Brescia, Italy). Cells were grown in 75 cm^2^ tissue flasks with DMEM medium supplemented with 100 U/mL penicillin, 100 μg/mL streptomycin, 2 mM L-glutamine, and 10% FBS under a humidified atmosphere of 5% CO_2_ at 37 °C. After colonies were formed (80%–90% of confluence), plates were washed with PBS 1X and harvested by 0.05% trypsin in 0.02% Na_4_EDTA for 5 min at 37 °C. For RT-PCR and immunoblotting experiments, cells were seeded into multiple-well plates or flasks at 1 x 10^5^ cells/mL concentration for 24 and 48 h. Then, the cells were treated with 100 and 200 nM VD3 or 3U and 6U nSMase after 24 h of seeding.

### 4.3. Cell Viability

MTT assay was used to test cellular viability, as previously reported [32]. HaCaT cells were seeded into 96-well plates at a density of 1 × 10^4^ cells/well with DMEM complete medium. After 24 h, culture medium was replaced with fresh complete medium containing different concentrations (10, 20, 40, 60, 80, 100, 120, 140, 160, 180, 200, 220, 240, and 300 nM) of VD3, and the cells were incubated for 24 and 48 h. Then, MTT reagent was dissolved in PBS 1X and added to the culture at 0.5 mg/mL final concentration. After 3 h incubation at 37 °C, the supernatant was carefully removed, and formazan salt crystals were dissolved in 200 µL DMSO that was added to each well. The absorbance (OD) values were measured spectrophotometrically at 540 nm using an automatic microplate reader (Eliza MAT 2000, DRG Instruments, GmbH, Marburg, Germany). Each experiment was performed two times in triplicate. Cell viability was expressed as a percentage relative to the control cells. The IC50 value was calculated by ED50plus Excel program.

### 4.4. Trypan Blue Exclusion Assay

After counting live cells, trypan blue exclusion assay was performed as previously reported [19] using a Countess™ (Invitrogen Srl, Milan, Italy) automated cell counter. Briefly, 50* *μL of VD3-treated HaCaT cell suspension (5 × 10^4^/500 μL) was mixed with equal volumes of 0.4% trypan blue and loaded onto a Countess cell-counting chamber slide. Untreated cells were used as control. The instrument was equipped with a camera that acquired images from cell samples on the chamber slide, and the image analysis software automatically analyzed the acquired cell images and measured cell count and viability.

### 4.5. In Vitro Wound Healing Assay

The wound healing assay was performed as previously reported [33] with CytoSelect™ Wound Healing Assay Kit (Cell Biolabs Inc., San Diego, CA, USA). HaCaT cell suspension (5 × 10^4^/500 μL) were seeded into 24-well tissue culture plate containing proprietary treated inserts in the plate wells with their “wound field” aligned in the same direction and incubated for 24 h to allow the cells to adhere and reach 80% confluence. After removing the inserts from the wells, the medium was carefully aspired. Then, the wells were washed twice with serum-free medium to remove dead cells and debris. Finally, the cells were treated with 100 and 200 nM VD3 for 6, 12, and 24 h. Untreated cells were used as control. To analyze cell migration, representative images focused on the center of the wound field were photographed according to manufacturer’s instructions. At least 3 fields for each condition were taken, and the numbers of migrating cells into scratched fields were calculated. Three sets of experiments in duplicates were performed. 

### 4.6. Quantitative Real-Time RT-PCR 

HaCaT cells cultured in the absence or presence of VD3 were used for total RNA extraction performed using RNAqueous^®^-4PCR kit (Ambion Inc., Austin, TX, USA). RTqPCR was performed as previously reported [34] using Master Mix TaqMan Gene Expression and 7.300 RT-PCR instrument (Applied Biosystems), targeting genes in TaqManArray 96-well plate P/N: 4414250, VDR (Hs00172113_m). Cyclin D1 (CCND1, HS00765553), cyclin-dependent kinase inhibitor 1A (CDK1A, Hs00355782), growth arrest and DNA-damage-inducible alpha (GADD45, Hs00169255_m1), phosphatase and TEN sinhomolog (PTEN, Hs02621230_S1), SM phosphodiesterase 1 (SMPD1, Hs03679347_g1), and SM phosphodiesterase 4 (SMPD4, Hs04187047_g1) genes were tested. Glyceraldehyde-3-phosphate dehydrogenase (GAPDH, Hs99999905_m1) and 18S rRNA (S18, Hs99999901_s1) were used as housekeeping genes. mRNA relative expression levels were calculated as 2^−ΔΔ*C*t^ by comparing the results of VD3-treated sample with those of untreated samples.

### 4.7. Western Blotting

About 30 μg of cell proteins were used for SDS-PAGE electrophoresis in 10% polyacrylamide slab gel. Proteins were transferred into nitrocellulose for 90 min as previously described [35]. The membranes were blocked for 30 min with 5% no-fat dry milk in PBS (pH 7.5) and incubated overnight at 4 °C with β-tubulin-specific antibody. The blots were treated with HRP-conjugated secondary antibodies for 90 min. Visualization was performed with the enhanced chemiluminescence kit from Amersham (Rainham, Essex, UK). The apparent molecular weight of proteins was calculated according to the migration of molecular size standards. The area density of the bands was evaluated by densitometry scanning and analyzed with ImageJ program.

### 4.8. Statistical Analysis

Three experiments in duplicate where performed for each analysis. The data are expressed as mean  ±  S.D., and the *t* test was used for statistical analysis.

## 5. Conclusions

In conclusion, wound repair can be obtained with average physiological levels of VD3 thanks to the VD3–nSMase cross-talk. We can speculate that insufficient VD3 concentration in the blood can slow down wound repair. The results from the study could be very useful in the future to improve therapeutic interventions for skin lesions.
